# Therapeutic inhibition of FcγRIIb signaling targets leukemic stem cells in chronic myeloid leukemia

**DOI:** 10.1038/s41375-020-0977-8

**Published:** 2020-07-20

**Authors:** Oliver Parting, Samantha Langer, Maja Kim Kuepper, Caroline Wessling, Shaoguang Li, Till Braunschweig, Nicolas Chatain, Tiago Maié, Ivan G. Costa, Martina Crysandt, Michael Huber, Tim H. Brümmendorf, Steffen Koschmieder, Mirle Schemionek

**Affiliations:** 1 Department of Hematology, Oncology, Hemostaseology, and Stem Cell Transplantation, Faculty of Medicine, University Hospital RWTH Aachen, Aachen, Germany; 2 Department of Transfusion Medicine, University Hospital Essen, Essen, Germany; 3 Department of Neurosurgery, Clemens Hospital, Muenster, Germany; 4 Department of Medicine, University of Massachusetts Medical School, Worcester, MA USA; 5 Department of Pathology, University Hospital RWTH Aachen, Aachen, Germany; 6 Institute for Computational Genomics, Joint Research Center for Computational Biomedicine, RWTH Aachen University, Aachen, Germany; 7 Institute of Biochemistry and Molecular Immunology, RWTH Aachen University, Aachen, Germany

**Keywords:** Cancer stem cells, Haematological cancer

## Abstract

Despite the successes achieved with molecular targeted inhibition of the oncogenic driver Bcr-Abl in chronic myeloid leukemia (CML), the majority of patients still require lifelong tyrosine kinase inhibitor (TKI) therapy. This is primarily caused by resisting leukemic stem cells (LSCs), which prevent achievement of treatment-free remission in all patients. Here we describe the ITIM (immunoreceptor tyrosine-based inhibition motif)-containing Fc gamma receptor IIb (FcγRIIb, CD32b) for being critical in LSC resistance and show that targeting FcγRIIb downstream signaling, by using a Food and Drug Administration-approved BTK inhibitor, provides a successful therapeutic approach. First, we identified FcγRIIb upregulation in primary CML stem cells. FcγRIIb depletion caused reduced serial re-plaiting efficiency and cell proliferation in malignant cells. FcγRIIb targeting in both a transgenic and retroviral CML mouse model provided in vivo evidence for successful LSC reduction. Subsequently, we identified BTK as a main downstream mediator and targeting the Bcr-Abl-FcγRIIb-BTK axis in primary CML CD34^+^ cells using ibrutinib, in combination with standard TKI therapy, significantly increased apoptosis in quiescent CML stem cells thereby contributing to the eradication of LSCs.. As a potential curative therapeutic approach, we therefore suggest combining Bcr-Abl TKI therapy along with BTK inhibition.

## Introduction

Chronic myeloid leukemia (CML) is caused by the Bcr-Abl translocation arising in the hematopoietic stem cell (HSC) compartment. The oncogenic Bcr-Abl tyrosine kinase induces a variety of signaling pathways that result in increased proliferation, differentiation, cytokine independence, and protection from apoptosis in the malignant cell clones [1–3]. Implementation of Bcr-Abl tyrosine kinase inhibitors (TKIs) showed remarkable clinical success with high response rates [4]. However, current therapies are often not curative, and even in patients responding optimally to Bcr-Abl inhibition, TKI discontinuation induces molecular relapse in about half of this patient cohort [5–7]. Bcr-Abl-positive HSCs are termed leukemic stem cells (LSCs) and this cell compartment contains quiescent cells that at least partially resist Bcr-Abl inhibition [8, 9]. Quiescent LSCs therefore differ from cycling CML cells that usually undergo apoptosis upon Bcr-Abl targeting [10, 11]. These resistant LSCs persist despite long-term TKI therapy in CML patients and are most likely the cause of disease recurrence upon TKI discontinuation. To increase the amount of patients achieving and remaining in treatment-free remission (TFR) therefore requires successful targeting of these LSCs.

Among many others, targeting JAK/STAT, IL-1, p53/MYC signaling, or metabolic activity has recently been shown to impair LSC survival [12–16]. Eventually, these observations have given rise to clinical trials, but successful implementation into routine clinical application could not be achieved so far.

ITIM (immunoreceptor tyrosine-based inhibition motif) receptors are classified as negative regulators, as they can recruit phosphatases and in this way inhibit signal transduction. The ITIM-receptor Fc gamma receptor IIb (FcγRIIb, CD32b) is a member of the Fc receptor family, which is known for their function to equalize signaling pathways and cell activation, leading to a well-balanced immune response [17]. FcγRIIb is most broadly expressed on all leukocytes except natural killer and T cells, is the only member transmitting inhibitory signals, and is predominantly phosphorylated by the SRC family kinase LYN [18]. FcγRIIb was previously identified as a cooperative oncogene upon disease progression in a preleukemic NUP98-HOXA9 mouse model [19]. Recently, ITIM motif containing receptors LAIR1 and PirB were identified to support acute myeloid leukemia (AML) stem cell survival [20, 21], highlighting the importance of those receptor types for LSC biology.

In this study, we identified TKI-independent upregulation of the ITIM-containing receptor FcγRIIb in primary murine leukemic stem and progenitor cells, as well as in primitive CML patient-derived CD34^+^ cells. Genetic depletion as well as short hairpin RNA (shRNA)-mediated knockdown impaired disease phenotype and LSC burden in vivo in a transgenic and retroviral CML mouse model. Subsequently, we identified the tyrosine kinase BTK as a main target of altered FcγRIIb downstream signaling, with BTK being activated only in FcγRIIb-expressing malignant cells. Implementation of ibrutinib (Ibr) to target BTK in combination with standard TKI therapy increased apoptosis in quiescent CML CD34^+^ cells providing a novel curative approach using a Food and Drug Administration (FDA)-approved drug, aiming to eradicate persisting LSCs.

## Materials and methods

### Isolation and cell culture of primary cells

Murine lin^−^ bone marrow (BM) cells were isolated and applied with or without HoxB8-driven immortalization that was performed as described [22, 23]. Mononucleated cells (MNCs) were purified by density gradient centrifugation from peripheral blood (PB), leukapheresis, or BM biopsy (Ficoll, GE Healthcare, Solingen, Germany), and then cultured in Iscove’s modified Dulbecco’s medium and 20% BIT9500 (Stem Cell Technologies). Isolation of CD34^+^ cells was performed from leukapheresis or BM biopsies using CliniMACS-CD34 or CD34-MicroBead Kit (human, Milteny Biotech, Bergisch Gladbach, Germany). For experimental procedures, cells were kept in low growth factor cocktail [9]. All cytokines were purchased from ImmunoTools (Friesoythe, Germany) or Milteny Biotech.

### Retroviral transduction

Retroviral transduction was performed as described previously [24] using CaCl_2_ for transfection and retronectin (Takara Bio Inc., France) for transduction. Transduction was repeated two to three times on consecutive days.

### RNA expression analysis

Differential expression analysis shown in Supplementary Fig. S17 was performed on the Gene Expression Omnibus dataset GSE2535. Data were acquired and analyzed using the GEOquery and limma R packages. Limma’s voom normalization and linear model fitting procedure were applied to the data in order to obtain the differentially expressed genes. We considered the origin of the samples (Leipzig vs. Mannheim) as a batch-effect covariate and corrected for such when fitting the model. As in GSE2535_REF, non-adjusted *p* values were used to identify the most differentially expressed genes. RNA extraction was performed using Trizol reagent (Thermo Fisher Scientific, Waltham, MA, USA). One microgram of total RNA was subjected to complementary DNA (cDNA) synthesis using the Moloney murine leukemia virus reverse transcriptase (Thermo Fisher). Gene expression was analyzed using TaqMan assays or SYBR Green method as described previously [25]. TaqMan Assays were purchased from Applied Biosystems (human FcγRIIb: Hs01634996_s1; murine FcγRIIb: Mm00438875_m1). Sequences for primer and probes are available in Supplementary Table S1.

### DNA constructs

FcγRIIb cDNA was amplified from cDNA of C57BL/6 wild-type BM using the following primer pairs: FcγRIIb_*Xho*I_F: GAACTCGAGATGGGAATCCTGCCGTTCCT and FcγRIIb_*Eco*RI_R: GAAGAATTCCTAAATGTGGTTCTGGTAAT. PCR product and MSCV-IRES-GFP vector were digested using *Xho*I and *Eco*RI. Positive clones were fully sequenced.

### FcγRIIb shRNA constructs

FcγRIIb targeting shRNA and scrambled (scr) control shRNA retroviral constructs were generated using pMSCV-LTR-miR30-SV40-GFP vectors. shRNA oligonucleotide sequences are available in Supplementary Table S2.

### Cell culture

32Dcl3 (named as 32D), K562, and KCL-22 (DSMZ, ACC 411, ACC 10, ACC 519) cells were cultured in RPMI media supplemented with 10–20% fetal bovine serum and 1% penicillin/streptomycin. Ten percent WEHI supernatant was used as IL-3 source.

### Colony formation assay

Indicated cell numbers were resuspended in methylcellulose (MethoCult M3231, M3534, H4230 and H4434, Stem Cell Technologies) and colony-forming units were counted after 7–9 days.

### Immunoblotting

Immunoblotting was performed as described previously [23]. The following antibodies were used for immunoblotting p27, pSTAT5Y694, STAT5, pCRKLY207; CRKL, pBTKY223, BTK, pERKY202/204; ERK, pp38Y180/182, p38, pcAbl, cAbl (Cell Signaling/New England Biolabs, Frankfurt, Germany), SHIP1 (N1), FcγRIIb (N16), GAPDH (Santa Cruz Biotechnology, Santa Cruz, CA, USA), GFP (Rockland, Limerick, PA, USA), pFcγRIIb (LifeSpan BioScience, Seattle, WA, USA), and Tubulin (Sigma-Aldrich, St. Louis, MO, USA). Protein detection was performed using Fusion SL chemoluminescence detector (PeqLab, Erlangen, Germany).

### Mice and genotyping

C567BL/6 FcγRIIb^−/−^ mice were purchased from Taconic (Cologne, Germany). Genotyping was performed using FcγRIIb_Neo_F:ATCGCCTTCTATCGCCTTCT; FcγRIIb_WT_F: TGCTTTACCCTGCTGAGGAT; FcγRIIb_WT_R: GGTCTGCTCCATTTGACACC enabling the identification of mutant FcγRIIb knockout (253 bp), wild-type (439 bp), or heterozygous (253 and 439 bp) mice. C567BL/6 wild-types were purchased from Janvier Labs (Genest-Saint-Isle, France). Genotyping of SCLtTA/Bcr-Abl mice was described previously [26]. FVB/N CD45.2 recipients were bred in-house.

### BM transplantation

BM cells from C567BL/6 FcγRIIb^−/−^, C567BL/6 FcγRIIb^+/+^ wild-type, and SCLtTA/Bcr-Abl mice were isolated 4 days after 5-FU injection. BM cells were retrovirally infected using MSCV-IRES-ev (empty vector)-GFP, MSCV-IRES-Bcr-Abl-GFP, MSCV-LTR-miR30-SV40-GFP-shRNA:scr, or MSCV-LTR-miR30-SV40-GFP-shRNA:FcγRIIb. Briefly, 1200 GFP^+^ Bcr-Abl-infected cells were transplanted together with 1 × 10^5^ wild-type rescue BM cells into 11 Gy irritated C567BL/6 wild-type recipients. Recipients were sacrificed for analyses 15 days after transplantation. A total of 2.5 × 10^6^ cells from LTR-miR30-SV40-GFP-shRNA:scr (shRNA:scr) and MSCV-LTR-miR30-SV40-GFP-shRNA:FcγRIIb (shRNA:FcγRIIb) non-fluorescence-activated cell sorting (FACS) sorted SCLtTA/Bcr-Abl BM cells were transplanted via tail vein injection into 10 Gy irradiated FVB/N CD45.2 mice. Mice were kept on cotrimoxazole (Ratiopharm, Ulm, Germany) for 1 week after transplantation.

### Ethics approval and consent to participate

Application of human samples was approved by the local ethics board of the medical faculty RWTH Aachen (EK127/12, EK206/09). Informed consent was obtained from all patients. All animal experiments were approved by the local authorities (Landesamt für Natur, Umwelt und Verbraucherschutz NRW, LANUV Az. 84-02.04.2013. A072).

### Flow cytometry analysis

BM and spleen cells from recipient mice were isolated as described before [26]. Antibodies for immunophenotyping applied in this study are available in Supplementary Table S3.

### Apoptosis assay

Apoptosis was analyzed using APC Annexin V Apoptosis Detection Kit (BioLegend) according to the manufacturer’s protocol.

### Analysis of cell cycle phase

Cell cycle analysis was performed using propidium iodide. Briefly, 2 × 10^6^ cells were fixed with cold ethanol and subsequently incubated with 100 µg/ml RNAse (Qiagen, Hilden, Germany) and 50 µg/ml propidium iodide (Sigma-Aldrich) for 30 min. Analysis was performed using FACS.

### Proliferation assay

Cell proliferation was assessed via Trypan blue exclusion method. Cell count was analyzed every 24 h for 4 days. To analyze proliferation of CML, CD34^+^ CellTraceTM CFSE Kit (Thermo Fisher Scientific) was used according to the manufacturer’s protocol.

### Preparation and storage of reagents

Imatinib (IM) (LC Laboratories, Woburn, MA, USA) was dissolved in dimethylsulfoxide (DMSO) and stored at −20 °C in stocks of 10 mM. Ibr (Selleckchem, Houston, TX, USA) was dissolved in DMSO at a stock concentration of 10 mM and stored at −80 °C.

### Statistical analysis

Statistical analyses were performed using GraphPad Prism software (La Jolla, CA, USA). FACS data were analyzed using FlowJo (Version 10) or Kalzua (Version 1.3) software. Student’s two-sided *t* test or Mann–Whitney *U* test were applied to compare the variability between two groups. Multiple group analyses were performed using one-way analysis of variance with Bonferroni’s multiple comparison test. *P* < 0.05 was considered as statistically significant. Error bars are given as standard derivation (s.d.).We performed neither blinding nor randomization within the animal experiments conducted in this study.

## Results

### Malignant FcγRIIb upregulation is not targeted by TKI therapy

**Fig. 1:**
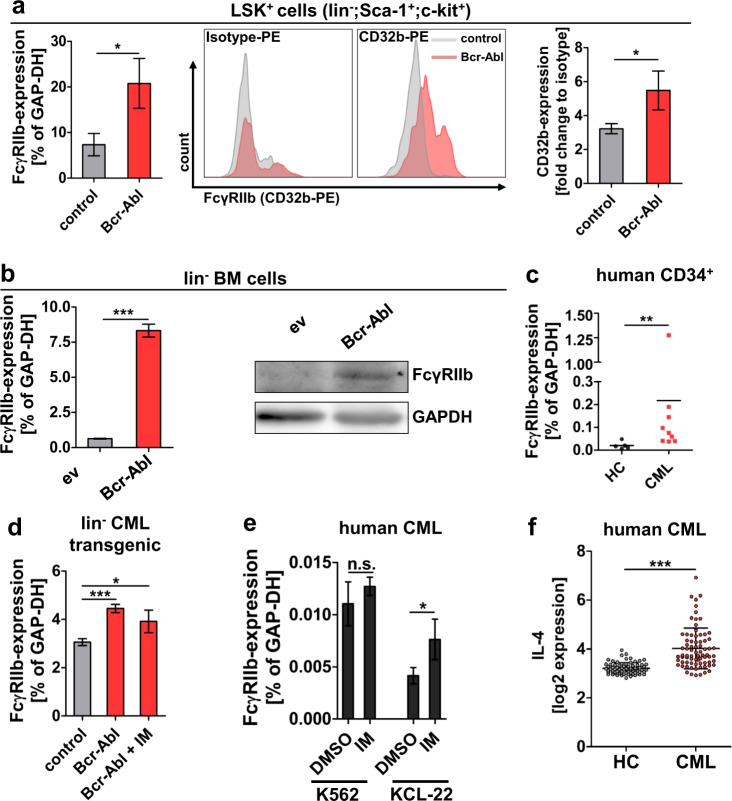
FcγRIIb is upregulated in murine and human leukemic stem cells. **a** FcγRIIb RNA and protein expression were analyzed in LSK^+^ cells (lin^−^;Sca-1^+^;c-kit^+^) from transgenic SCLtTA/Bcr-Abl mice that had been induced to express Bcr-Abl vs. controls. FACS sorted LSK^+^ from 3 weeks induced mice were analyzed using qRT-PCR (*n* = 3/3). The cell surface expression of FcγRIIb (CD32b) was assessed by FACS in mice that had been induced for 6 days (*n* = 3/3). **b** Lineage-depleted BM cells from C567B/L6 wild-type mice were virally transduced to express Bcr-Abl or empty vector (ev) control. Transduced cells were FACS sorted and analyzed for FcγRIIb expression using qRT-PCR (left) and western blot analysis (right). **c** FcγRIIb mRNA expression was analyzed in CD34^+^ healthy control (HC) and CML patient samples (*n* = 5/9). **d** Lineage negative (lin^−^) BM cells from transgenic SCLtTA/Bcr-Abl mice vs. controls were analyzed for FcγRIIb expression before and after 2 µM imatinib (IM) treatment for 18 h. **e** K562 and KCL-22 cells were treated for 18 h with 5 µM imatinib. **f** Public available microarray data from the leukemia MILE study (#GSE13159 and #GSE13164, probe set id: 207539_s_at) from BM biopsy of healthy control (HC) and CML patients at first diagnosis (CML) were analyzed for IL-4 expression (*n* = 73/76). Data are shown as mean ± SD. *p < 0.05, **p < 0.001, ***p < 0.0001, and n.s. not statistically significant.

We previously identified upregulation of FcγRIIb (CD32b) in LSK (lin^−^;c-kit^+^,Sca-1^+^) cells from transgenic SCLtTA/Bcr-Abl CML-CP mice (2.8-fold, *p* < 0.05) by microarray analysis [26], and here we first confirmed upregulation of the receptor by real-time quantitative PCR (qRT-PCR) (FcγRIIb) and FACS analysis (CD32b) in these malignant LSK cells (Fig. 1a). Next, we studied murine C567BL/6 lin^−^ BM cells that were virally transduced to express Bcr-Abl. FcγRIIb messenger RNA (mRNA) and protein levels were again found to be significantly elevated in Bcr-Abl^+^ cells vs. ev controls (Fig. 1b). Finally, we tested FcγRIIb mRNA expression in CML vs. normal CD34^+^ cells and observed a 10.7-fold increase in the human progenitor cell population (Fig. 1c). We proceeded to examine if TKI treatment could revert malignant FcγRIIb upregulation. CML cells from transgenic mice (Fig. 1d) and human CML cell lines K562 and KCL-22 (Fig. 1e) were treated with IM and this showed persisting FcγRIIb expression; TKI treatment even enhanced FcγRIIb mRNA expression in the latter cell line. As it has been shown that FcγRIIb expression is increased by the anti-inflammatory cytokine IL-4 [27], we analyzed publicly available microarray data for IL-4 expression in CML patients (GSE13159 and GSE13164, probe set id: 207539_s_at). IL-4 expression was significantly increased in CML vs. normal samples (Fig. 1f). In agreement, addition of IL-4 was able to increase FcγRIIb mRNA expression levels in the CML cell line KCL-22, and combined treatment with TKI could not antagonize elevated IL-4 expression, but even enhanced this effect (Supplementary Fig. S1), suggesting that TKI-persisting malignant upregulation could be mediated via altered cytokine levels in CML.

### FcγRIIb depletion impairs leukemic stem and progenitor cell function

**Fig. 2:**
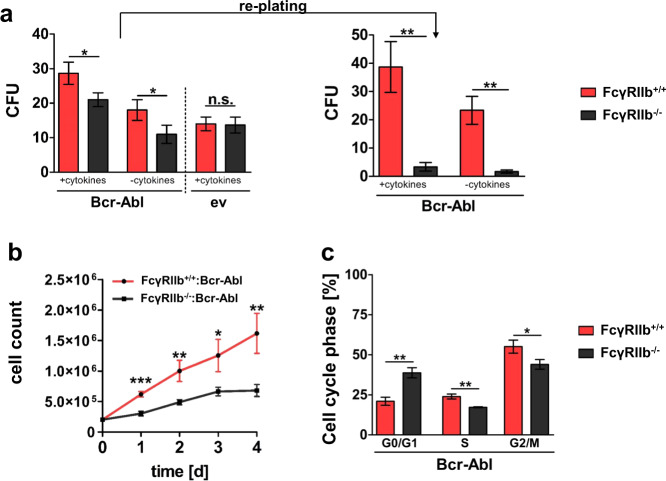
FcγRIIb knockout reduces leukemic clonogenic potential and cell proliferation. **a** Bcr-Abl and ev (empty vector) infected lin^−^ BM cells from FcγRIIb^+/+^ and FcγRIIb^−/−^ (1000 cells/ml) were seeded in methylcellulose with or without cytokines. Numbers of colonies (colony-forming units, CFUs) were assessed after 7 days. Re-plating was performed using 1 × 10^4^ cells. CFU numbers were counted after 7 days. **b** Proliferation assay using FcγRIIb^+/+^ and FcγRIIb^−/−^ Bcr-Abl^+^ cells was performed using Trypan blue exclusion method. **c** Cell cycle was assessed in FcγRIIb^+/+^ and FcγRIIb^−/−^ Bcr-Abl^+^ cells. Data are shown as mean ± SD. *n* = 3, each, *p < 0.05, **p < 0.001, ***p < 0.0001, and n.s. not statistically significant.

In order to study the effect of receptor targeting in malignant cells, we next applied lin^−^ BM cells from FcγRIIb^+/+^ and FcγRIIb^−/−^ mice that were virally infected to express Bcr-Abl or ev control. Colony-forming unit (CFU) assays using Bcr-Abl^+^ or ev lin^−^ FcγRIIb^+/+^ and FcγRIIb^−/−^ cells revealed significantly decreased colony numbers upon receptor depletion specifically in Bcr-Abl^+^ cells (Fig. 2a). Moreover, CFU numbers were strongly reduced upon re-plating, suggesting reduced stem cell function of leukemic FcγRIIb^−/^^−^ cells (Fig. 2a). As lin^−^ cells represent a rare cell population, we immortalized stem and progenitor cells using an estradiol regulated Hoxb8 approach [22]. We confirmed reduced clonogenic potential upon FcγRIIb knockout likewise in these cells (Supplementary Fig. S2). Next, we analyzed cell proliferation from immortalized leukemic FcγRIIb^+/+^ vs. FcγRIIb^−/−^ cells. FcγRIIb knockout significantly impaired proliferation in Bcr-Abl positive (Fig. 2b), but not Bcr-Abl-negative control cells (Supplementary Fig. S3). We proceeded to perform cell cycle analysis. Deficiency of FcγRIIb increased the G0/G1 cell fraction, while S and G2/M phases were decreased (Fig. 2c). No cell cycle alterations were observed in ev control cells (Supplementary Fig. S4). Analyses of negative cell cycle regulator p27^Cip1^ revealed increased protein expression in leukemic FcγRIIb knockout cells (Supplementary Fig. S5). Moreover, we observed increased RNA expression of the negative cell cycle regulators p16^Ink4A^, p19^Arf^, and p27^Cip1^, but not p21^Cip/Waf1^, upon FcγRIIb depletion in Bcr-Abl-positive cells (Supplementary Fig. S6). Taken together, these data show impaired malignant, but not normal stem and progenitor cell function upon FcγRIIb targeting.

### FcγRIIb knockout or knockdown impairs CML development in vivo

**Fig. 3:**
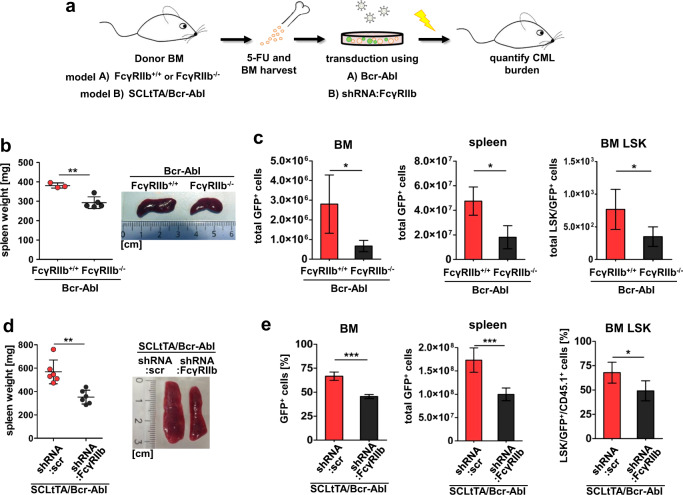
Loss or reduction of FcγRIIb attenuates CML development and reduces leukemic LSK cells in vivo. **a** Schematic illustration of bone marrow transplantation approach using either FcγRIIb^+/+^ and FcγRIIb^−/−^ C567B/L6 cells that were virally transduced to express Bcr-Abl (model A) or SCLtTA/Bcr-Abl FVB/N-derived BM cells that were transduced to express shRNA targeting FcγRIIb (model B). **b** Spleen weight of transplanted recipients receiving FcγRIIb^+/+^:Bcr-Abl (*n* = 3) or FcγRIIb^−/−^:Bcr-Abl (*n* = 5) BM cells. **c** Total leukemic GFP^+^ cells in BM, spleen, and BM LSK compartment. **d** Spleen weight of FVB/N recipient mice transplanted with SCLtTA/Bcr-Abl BM cells that were transduced with either shRNA:scr control or shRNA:FcγRIIb. **e** Total leukemic GFP^+^ cells in BM, spleen, and BM LSK compartment. Data are shown as mean ± SD. *p < 0.05 and **p < 0.001.

In order to study FcγRIIb function in vivo, we applied both a Bcr-Abl transduction model combined with genetic receptor depletion (Fig. 3a, model A) as well as strongly reduced FcγRIIb expression mediated by shRNA using the transgenic SCLtTA/Bcr-Abl CML mouse model (Fig. 3a, model B). For the retroviral model, we transplanted FcγRIIb^+/+^ or FcγRIIb^−/−^ Bcr-Abl-transduced BM cells into syngeneic C57BL/6 recipients. C57BL/6 mice receiving FcγRIIb^−/−^ Bcr-Abl-infected BM cells revealed a significantly reduced white blood cell compared to FcγRIIb^+/+^ Bcr-Abl-transplanted recipients (Supplementary Fig. S7). Spleen weight of recipients was analyzed upon autopsy 15 days after transplantation revealing a significant reduction upon FcγRIIb depletion (Fig. 3b). Vector-encoded GFP expression allowed for FACS analysis of Bcr-Abl-positive leukemic cells and revealed a reduction in total BM (4.19-fold) and spleen (2.62-fold) upon transplantation of FcγRIIb^−/−^ Bcr-Abl compared to FcγRIIb^+/+^ Bcr-Abl BM (Fig. 3c). Moreover, FcγRIIb depletion reduced leukemic LSK cells by 2.19-fold (Fig. 3c). Next, we applied shRNA-mediated knockdown of FcγRIIb in vitro and in vivo using the SCLtTA/Bcr-Abl transgenic CML mouse model (Fig. 3a, model B).

Therefore, we first determined FcγRIIb knockdown efficiency on mRNA and protein level using different shRNAs (Supplementary Fig. S8). We identified shRNA#3 that showed the strongest knockdown on protein expression and proceeded to reduce FcγRIIb expression using this shRNA; hereafter, named shRNA:FcγRIIb. We first assessed proliferation of immortalized SCLtTA/Bcr-Abl BM cells and this was significantly impaired due to FcγRIIb knockdown (Supplementary Fig. S9). Likewise, clonogenic potential was drastically reduced upon FcγRIIb knockdown (9.95-fold, Supplementary Fig. S10). We then transplanted shRNA:FcγRIIb- or scr:shRNA-transduced control BM cells from SCLtTA/Bcr-Abl (CD45.1) into FVB/N (CD45.2) mice. Recipients were sacrificed 18 days after transplantation for analyses. Spleen weight was assessed upon autopsy and revealed a significant reduction in SCLtTA/Bcr-Abl shRNA:FcγRIIb-transplanted recipients compared to SCLtTA/Bcr-Abl shRNA:scr control mice (Fig. 3d). shRNA-infected cells were identified via the vector-encoded GFP expression and were reduced in BM (1.42-fold) and spleen (1.73-fold) upon FcγRIIb knockdown (Fig. 3e). Next, we analyzed LSK cells in this model revealing a 1.38-fold decrease upon FcγRIIb targeting (Fig. 3e). These effects were less severe as compared to complete depletion of the receptor (Fig. 3c), likely reflecting residual FcγRIIb expression/activity in the knockdown vs. knockout model.

### Malignant FcγRIIb signaling mediates BTK activation in leukemic progenitors

**Fig. 4:**
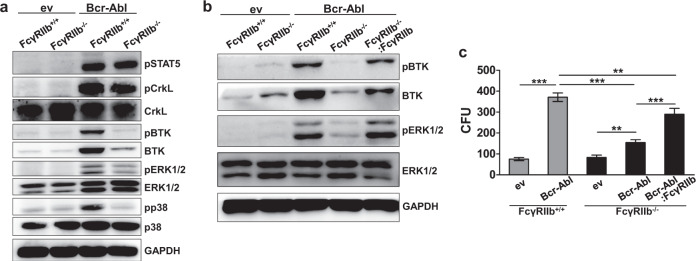
FcγRIIb mediates malignant BTK activation in Bcr-Abl-positive cells. **a** Western blot analyses of immortalized FcγRIIb^+/+^ and FcγRIIb^−/−^ Bcr-Abl and ev (empty vector) BM cells were performed using the indicated antibodies. **b** FcγRIIb was retrovirally introduced into FcγRIIb^−/−^:Bcr-Abl cells and protein lysates were analyzed for BTK and ERK1/2 activation. **c** CFU assay using FcγRIIb^+/+^:ev, FcγRIIb^−/−^:ev, FcγRIIb^+/+^:Bcr-Abl, FcγRIIb^−/−^:Bcr-Abl, or FcγRIIb^−/−^:Bcr-Abl:FcγRIIb cells. Colony numbers were counted 7 days after seeding. Data are shown as mean ± SD. **p < 0.001 and ***p < 0.0001.

We next studied the role of FcγRIIb-induced signal transduction employing, the immortalized FcγRIIb^−/−^ and FcγRIIb^+/+^ progenitor cell model. As expected, pSTAT5^Y694^ and pCRKL^Y207^ were present in leukemic FcγRIIb^−/−^ and FcγRIIb^+/+^ Bcr-Abl positive cells (Fig. 4a). Interestingly, we identified reduced phosphorylation levels of Bruton’s tyrosine kinase (BTK^Y223^), ERK1/2^Y202/204^ and p38^Y180/182^ in leukemic cells that were depleted for FcγRIIb. BTK total protein was likewise decreased. To analyze if reduced BTK signaling is truly FcγRIIb-dependent in Bcr-Abl-positive cells, we transduced FcγRIIb^−/−^ BM using an FcγRIIb-encoding virus. Western blot analyses confirmed that pBTK was largely restored upon re-introduction of the receptor in FcγRIIb^−/−^ cells (Fig. 4b). As we observed reduced CFU potential upon FcγRIIb depletion in malignant cells (Fig. 2a), we proceeded to test the effect of FcγRIIb restoration also on the clonogenic potential. Upon re-integration of FcγRIIb into leukemic knockout cells, CFU numbers significantly increased again (Fig. 4c). Leukemic FcγRIIb^−/−^ cells tended to form smaller and more diffuse colonies, compared to leukemic FcγRIIb^+/+^ cells. This effect was dependent on FcγRIIb, as cells with restored receptor expression again formed colonies similar to leukemic FcγRIIb^+/+^ cells (Supplementary Fig. S11).

### Inhibition of Bcr-Abl combined with Ibr therapy reduces malignant potential and enhances apoptosis in nondividing CML-CP LSCs

**Fig. 5:**
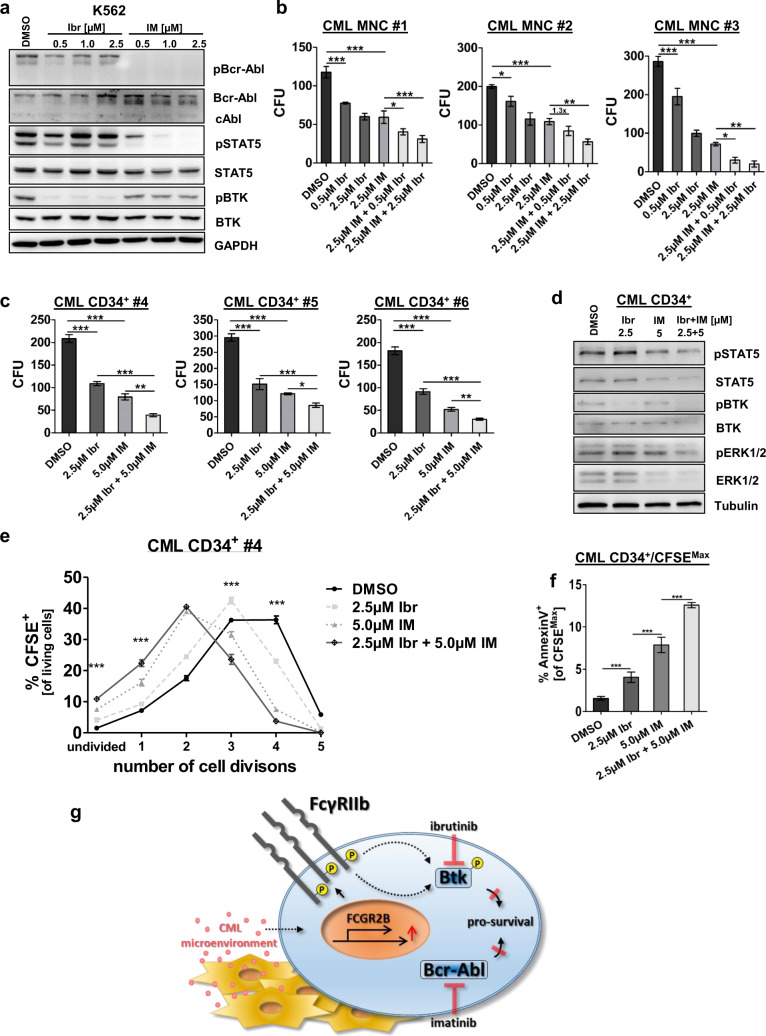
Combined inhibition of Bcr-Abl and BTK impairs clonogenic potential, reduces proliferation, and enhances apoptosis in nondividing LSCs. **a** K562 cells were treated with increasing concentrations of Ibr (Ibrutinib, 0.5–2.5 µM) or IM (Imatinib, 0.5–2.5 µM) for 18 h. Subsequently, protein lysates were analyzed for pBcr-Abl, pSTAT5, pBTK, Bcr-Abl, STAT5, BTK, and GAPDH. **b** Primary CML MNCs (10,000 cells/ml) were treated for 48 h (**c**) and CML CD34^+^ cells (1000 cells/ml) for 72 h with Ibr (0.5 µM, 2.5 µM), IM (2.5 µM, 5.0 µM), or the combination of both drugs. Cells were resuspended and plated using methylcellulose-containing cytokines. CFU numbers were counted after 7 days (*n* = 3, each). **d** CML CD34^+^ cells were treated for 72 h with 2.5 µM Ibr, 5.0 µM IM, and the combination of both drugs. Protein lysates were analyzed for pSTAT5, pBTK, pERK1/2, STAT5, BTK, and ERK1/2. **e** Evaluation of cell divisions in CML CD34^+^ cells upon treatment using CFSE combined with FACS analyses. For a clear presentation, the level of significance is given for Ibr + IM vs. IM only. **f** Annexin V^+^ staining of undivided CFSE^Max^ cells in treated CML CD34^+^ cells. **g** Proposed mechanism: elevated FcγRIIb expression in malignant cells persists despite BCR-ABL inhibition and this could be mediated via inflammatory cytokines in the CML microenvironment. FcγRIIb activity results in increased BTK expression and phosphorylation and dual targeting of BCR-ABL and BTK activity induces apoptosis in LSCs. Data are shown as mean ± SD. *p < 0.05, **p < 0.001, ***p < 0.0001, and n.s. not statistically significant.

As we identified BTK as a downstream target of FcγRIIb signaling in murine CML cells, we proceeded to study BTK phosphorylation in Bcr-Abl-positive K562 cells upon TKI treatment. As expected, Ibr treatment significantly decreased pBTK, but did not affect Bcr-Abl-induced signaling (Fig. 5a). In contrast, IM treatment abolished pBcr-Abl and pSTAT5 levels, but only mildly reduced pBTK providing a rationale for dual targeting. As it has been reported that Ibr treatment reduces Bcr-Abl kinase activity in a murine progenitor cell line [28], we first treated 32D Bcr-Abl-expressing cells. In line with these results, we observed reduced Bcr-Abl phosphorylation upon increasing Ibr concentration in the murine cell line model (Supplementary Fig. S12). However, in human CML Ibr treatment had only mild effects on pBcr-Abl and this was not enhanced even by increasing concentrations up to 2.5 µM (Fig. 5a). Next, we evaluated the effect of combined BTK and Bcr-Abl inhibition on MNCs and CML CD34^+^ cells. MNCs showed reduced CFU capacity upon IM and interestingly also upon Ibr treatment alone and administered together both drugs further reduced CFU numbers (Fig. 5b). Likewise, in CML CD34^+^ cells Ibr treatment significantly decreased colony formation compared to DMSO (Fig. 5c). Combined Ibr and IM therapy further reduced colony numbers compared to single TKI treatment in all samples tested (Fig. 5c). Protein expression analysis of CML CD34^+^ cells revealed that Ibr inhibited BTK phosphorylation, while STAT5 activation was not affected, confirming that Bcr-Abl downstream signaling remained unaffected by Ibr. Also, IM treatment alone did not affect pBTK levels (Fig. 5d). Combined BTK and Bcr-Abl inhibition reduced proliferation of TKI-insensitive quiescent (CFSE^Max^) CD34^+^ CML stem cells, yielding more cells with no or one division as compared to single IM treatment (Fig. 5e, Supplementary Figs. S13 + S14). However, apoptosis FACS of nondividing CFSE^Max^ CD34^+^ cells revealed a significant increase in the quiescent CML cell population (Fig. 5f, Supplementary Figs. S15 + S16), showing that combined treatment not only reduces the stem cell quality of CML cells, but also induces apoptosis in this therapy-resistant cell population. In conclusion, Ibr as an FDA-approved drug could therefore provide a novel therapeutic approach to overcome resistance in CML stem cells.

## Discussion

A variety of clinical trials has revealed that TKI discontinuation in optimal responding patients allows for TFR in about half of this selected patient cohort. Thereby, a minimum of 2 years of continued therapy in deep molecular response seems to be beneficial [5, 29], but beyond that the effect on TFR is largely unchanged either by further therapy duration or the type of TKI tested [6, 30, 31], showing that Bcr-Abl-independent mechanisms must be targeted to increase TFR rates in patients, ideally using already approved drugs.

Here we show a critical function of the inhibitory ITIM-receptor FcγRIIb in CML stem cell persistence and therapeutic targeting of malignant FcγRIIb-mediated signaling enhanced TKI-mediated cell death in CML stem cells.

A positive role of ITIM-receptor signaling for HSC function in AML has previously been shown. The ITIM-receptor LIRB2 and its mouse homologous PirB are expressed on human and mouse HSCs, where their binding to angiopoietin-like proteins supports stem cell expansion. Genetic depletion of PirB enhanced differentiation and impaired MLL-AF9 AML development in vivo and LSC survival [20, 32]. In ALL activation of the ITIM-receptor platelet endothelial cell adhesion molecule 1 (PECAM-1), CD300A, and LAIR1 has been shown to compensate for excessive signaling in B cells that is mediated through recruitment of the inhibitory phosphatases PTPN6 and INPP5D [33]. In CML the role of ITIM-receptor signaling has hardly been studied so far. Using human cell lines, the ITIM-receptor PECAM-1 was found to antagonize IM-induced apoptosis [34], but genetic depletion of PECAM-1 in a retroviral model of CML-like disease showed no effect on CML myeloid progenitor viability or clonogenic potential in vitro [33].

Here we at first identified TKI-resistant FcγRIIb upregulation in murine and human CML stem and progenitor cells. Cytokines such as IL-4 or IL-10 are known to induce FcγRIIb upregulation [27, 35] and, indeed, we could confirm IL-4-mediated FcγRIIb upregulation in malignant cells that was not decreased upon TKI treatment. Concordantly, expression of the IL-4 receptor alpha chain was significantly upregulated (2.23-fold, *p* = 0.0062, data not shown) in CML vs. normal LSK cells in our microarray analysis of the transgenic SCLtTA/Bcr-Abl mouse model [26], which likewise showed FcγRIIb upregulation. IL-4 was previously found to be increased in the plasma of CML patients [36] and corresponding to this IL-4 secretion by the malignant clone has been shown for K562 and BA/F3:Bcr-Abl CML cells, where IL-4 secretion antagonized IM-induced apoptosis [37]. Moreover, downregulation of IL-4 is evident in unfractionated BM or pB leukocytes from cytogenetic responders vs. non-responders [38] (Supplementary Fig. S17), and taken together, these data suggest that IL-4 could be a candidate for permitting TKI-persisting FcγRIIb upregulation. Further analyses correlating IL-4 serum levels with pBTK ideally in LSCs from patients who remain in or not remain in TFR after TKI discontinuation would (1) further underline the significance of pBTK in LSC resistance and (2) could unravel the potential of IL-4 or pBTK being a biomarker that would allow predicting the outcome of TKI discontinuation.

By using FcγRIIb^−/−^ BM cells, we identified markedly decreased clonogenic potential, particularly apparent upon serial re-plating, suggesting that primitive hematopoietic cells are notably affected by FcγRIIb targeting. Colonies that were generated by Bcr-Abl-positive FcγRIIb^−/−^ cells were less compact and more diffuse compared to Bcr-Abl-positive FcγRIIb^+/+^ cells, and more diffuse colonies indicate an increased differentiation potential [39]. Decreased CFU potential and colony morphology could be largely rescued by reconstituting FcγRIIb expression. Transplantation of FcγRIIb^−/−^ BM cells into recipient mice impaired the CML phenotype, including a reduction of leukemic LSK cells using a retroviral and transgenic CML mouse model. Reduced malignant cell expansion is in line with decreased proliferation in malignant FcγRIIb*-*depleted or SCLtTA/Bcr-Abl FcγRIIb shRNA-transduced cells. Moreover, we observed a strong increase in negative cell cycle regulators p16^Ink4A^, p19^Arf^, and p27^Cip1^ in leukemic FcγRIIb^−/−^ cells. These effects were only evident in malignant, but not in Bcr-Abl-negative FcγRIIb^−/−^ cells. Activation of cell cycle checkpoint molecules due to ITIM-receptor knockout was previously reported in Bcr-Abl-positive pre-B cells [33]. In this model, ITIM-receptor depletion resulted similarly in cell cycle arrest and complete loss of CFU potential.

The FcγRIIb ITIM motif can be phosphorylated by SRC family kinase LYN [18]. It has been shown that LYN-mediated FcγRIIb phosphorylation results preferentially in recruitment of SHIP1 (*INPP5D*) in vivo [40–42]. SHIP1 then hydrolyses 5′-phosphate of phosphatidylinositol-3,4,5-trisphosphate, which alters the interaction of various signaling molecules with the plasma membrane, thereby affecting intracellular signal transduction. In contrast to published data where SHIP1-dependent FcγRIIb signaling attenuates ERK1/2 activation [43], we observed reduced ERK1/2 phosphorylation due to FcγRIIb deficiency in leukemic cells. Similarly, we observed diminished BTK activation due to FcγRIIb knockout in malignant cells that could be restored upon rescued FcγRIIb expression. Global phosphotyrosine profiling previously identified an activated BTK-derived peptide in Bcr-Abl-expressing BA/F3 cells [44]. Applying the BTK inhibitor LFM-A13 on Bcr-Abl^+^ cell lines reduced cell viability similar to IM [45]. A further report studying BTK function by pharmacologic inhibition using PC-005 to inhibit BTK likewise showed reduced growth of a Bcr-Abl^+^ murine BA/F3 lymphoid progenitor cell line, but this was associated with off-drug target effects reducing Bcr-Abl kinase activity [28]. We have observed similar reduction of pBcr-Abl upon BTK inhibition using Ibr in murine Bcr-Abl-expressing 32D cell line. However, in human K562 CML cells as well as patient-derived CML CD34^+^ cells, no drastically altered Bcr-Abl or STAT5 phosphorylation was evident upon Ibr treatment.

Our data show elevated BTK protein expression in Bcr-Abl-positive transgenic cells, in the presence of FcγRIIb. Upon depletion of the receptor, BTK protein significantly decreases in malignant cells and this is partially restored by reconstituting FcγRIIb expression. Expression of total ERK or p38 protein is not affected by FcγRIIb depletion. Despite, phosphorylation of these targets is Bcr-Abl dependent, as expected. To this end, it has been shown that inhibition of Bcr-Abl kinase activity by IM significantly decreases phosphorylation of ERK1/2 or p38, while total protein remained unaffected [46]. Moreover, in a model of doxycycline-regulated Bcr-Abl expression, reversion of Bcr-Abl altered the level of ERK1/2 or p38 phosphorylation, while the total protein did not change [47]. However, the underlying mechanism that results in BTK protein reduction upon FcγRIIb depletion in malignant cells is unclear. It has been shown that BTK can induce its own transcription by a nuclear factor-κB (NF-κB)-dependent mechanism [48]. In addition, the FcγRIIb itself is induced upon NF-κB stimulation [49]. Along these lines, NF-κB is activated in CML cells despite Bcr-Abl inhibition, downstream of, for example, IL-1 [15] or TNF signaling [50]. Moreover, activation of NF-κB downstream of IL-4 has been shown [51] and our data already suggest that IL-4 is involved in TKI-resistant FcγRIIb upregulation (Fig. 1f and Supplementary Fig. S1). Interestingly, the deletion of the γ-chain subunit in FcγR knockout mice blocked NF-κB activation in these mice [52]. Therefore, it is tempting to speculate that BTK protein could be upregulated via NF-κB in malignant FcγRIIb-expressing cells and it would be highly interesting to study the effect of FcγRIIb depletion on NF-κB-mediated BTK activation in our model.

Simultaneous administration of Ibr and IM was superior in reducing the clonogenic potential of CML-CP patient-derived MNCs and primitive CD34^+^ cells compared to single TKI application. Moreover, Bcr-Abl inhibition combined with Ibr treatment increased apoptosis in quiescent CML-CP LSCs.

The BTK inhibitor Ibr is already approved for the treatment of CLL and mantle cell lymphoma and was further effective in pre-clinical AML studies, multiple myeloma, and pre-BCR B-ALL [53–55]. It has to be considered that Ibr therapy is associated with considerable toxicity, including atrial fibrillation, bleeding, neutropenia, and infections, as well as gastrointestinal symptoms [56–58]. Careful selection of patients and dosing will be important for phase I and II combination trials. In addition, given the current cost of Ibr, the potential treatment regime should be carefully elaborated. We could envision short-term Ibr treatment in patients who are already eligible for TKI discontinuation due to deep molecular remission. In these patients, Ibr could either replace Bcr-Abl-directed TKI therapy or be administered together with Bcr-Abl inhibition for a defined period. Molecular monitoring in close intervals during and after therapy could then unravel if TFR rate could be increased using this strategy. If toxicity is acceptable, the cost at long term would be reduced if a higher rate of patients will obtain TFR.

This study shows that BTK inhibition by the FDA-approved drug Ibr significantly affects LSC survival in CML and hence targeting the Bcr-Abl–FcγRIIb–BTK axis is a novel therapeutic strategy contributing to the eradication of therapy-resistant LSCs.

## Supplementary information

Supplementary Figures

Supplementary Information Tables
