# Supplementary material for: Therapeutic inhibition of FcγRIIb signaling targets leukemic stem cells in chronic myeloid leukemia
Source: Leukemia. 2020 Jul 20;34(10):2635–47. doi: 10.1038/s41375-020-0977-8 (PMC7515845; doi:10.1038/s41375-020-0977-8)
Supplement: Supplementary file 1 — Supplementary Figures [file 41375_2020_977_MOESM1_ESM.pdf]

Supplementary Information

Therapeutic inhibition of FcγRIIb signaling targets leukemic stem cells in chronic myeloid leukemia

Oliver Parting[1], Samantha Langer[2], Maja Kim Kuepper[1], Caroline Wessling[3], Shaoguang Li[4], Till Braunschweig[5], Nicolas Chatain[1], Tiago Maié[6], Ivan G. Costa[6] Martina Crysandt[1], Michael Huber[7], Tim H Brümmendorf[1], Steffen Koschmieder[1], and Mirle Schemionek[1]

[1]Department of Hematology, Oncology, Hemostaseology, and Stem Cell Transplantation, Faculty of Medicine, University Hospital RWTH Aachen, Aachen, Germany; [2]Department of Transfusion Medicine, University Hospital Essen, Essen, Germany; [3]Department of Neurosurgery, Clemens Hospital, Muenster, Germany; [4]Department of Medicine, University of Massachusetts Medical School, Worcester, Massachusetts, USA; [5]Department of Pathology, University Hospital RWTH Aachen, Aachen, Germany; [6]Institute for Computational Genomics, Joint Research Center for Computational Biomedicine, RWTH Aachen University, Aachen, Germany, [7]Institute of Biochemistry and Molecular Immunology, RWTH Aachen University, Aachen, Germany

Supplementary Figure S1

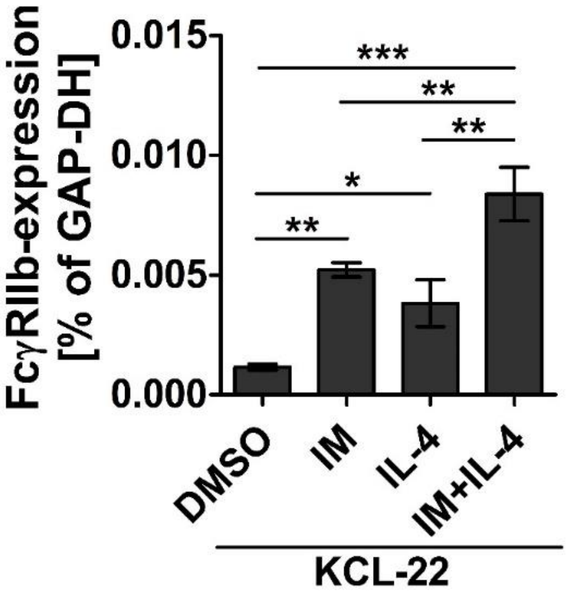

IL-4 contributes to malignant FcγRIIb upregulation in Bcr-Abl⁺ cells. CML KCL-22 cells were treated with 2µM Imatinib, 100ng/ml IL-4 or the combination. mRNA expression of FcγRIIb was evaluated by qRT-PCR. Data are shown as mean ± SD. $^{*}p<0.05$, $^{**}p<0.001$, $^{***}p<0.0001$

Supplementary Figure S2

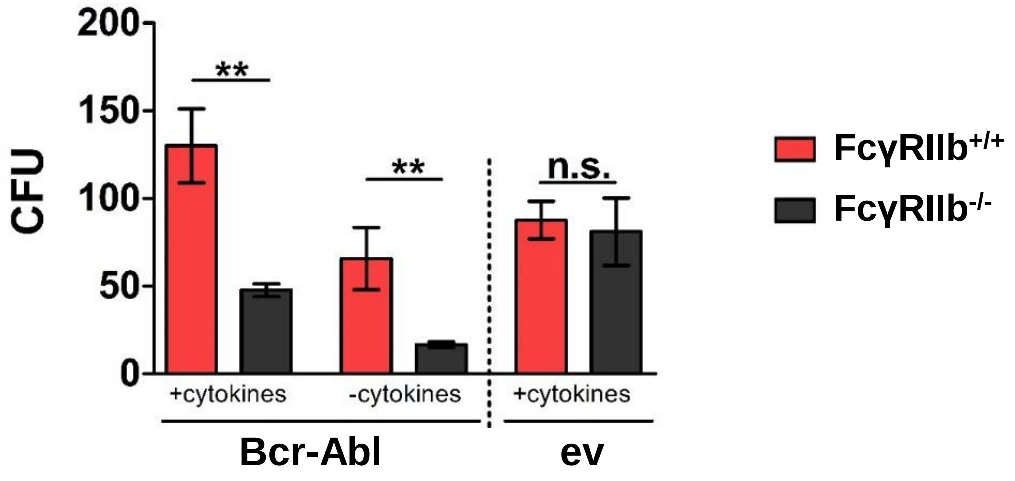

Clonogenic potential of malignant but not normal Hoxb8-immortalized FcγRIIb$^{-/-}$ cells is impaired. 2000 HoxB8-immortalized FcγRIIb$^{+/+}$:ev, FcγRIIb$^{-/-}$:ev, FcγRIIb$^{+/+}$:Bcr-Abl and FcγRIIb$^{-/-}$:Bcr-Abl BM cells were seeded in methylcellulose with and without cytokines and analyzed for colony formation after 7 days.

Supplementary Figure S3

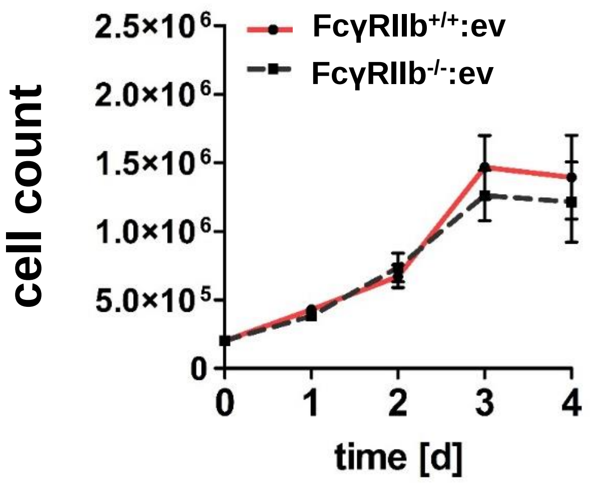

BM cell proliferation is not affected by FcγRIIb depletion. Proliferation of FcγRIIb$^{+/+}$:ev (empty vector) and FcγRIIb$^{-/-}$:ev transduced cells was analyzed using trypan-blue exclusion method.

Supplementary Figure S4

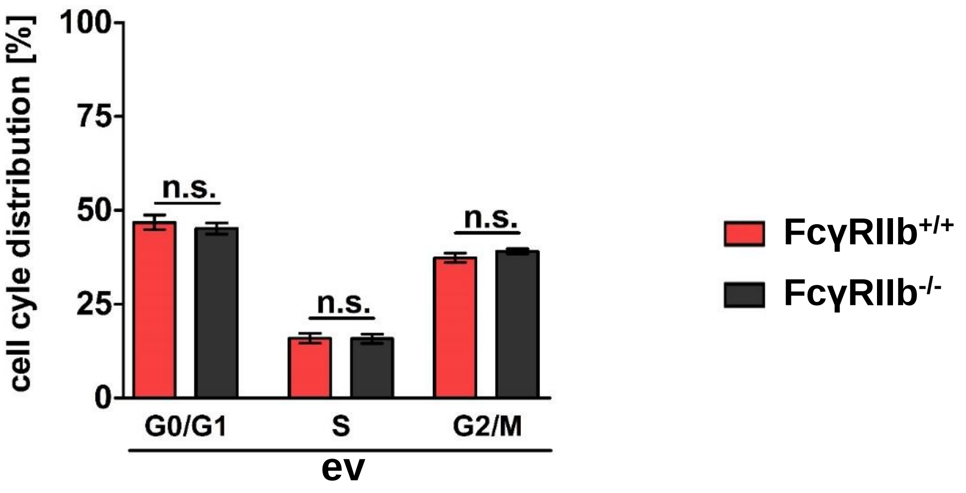

Cell cycle phase is not affected by FcγRIIb depletion. Cell cycle analysis of FcγRIIb$^{+/+}$:ev and FcγRIIb$^{-/-}$:ev BM cells was performed by propidium iodide method using FACS analysis. Data are shown as mean ± SD; ns= not statistically significant

Supplementary Figure S5

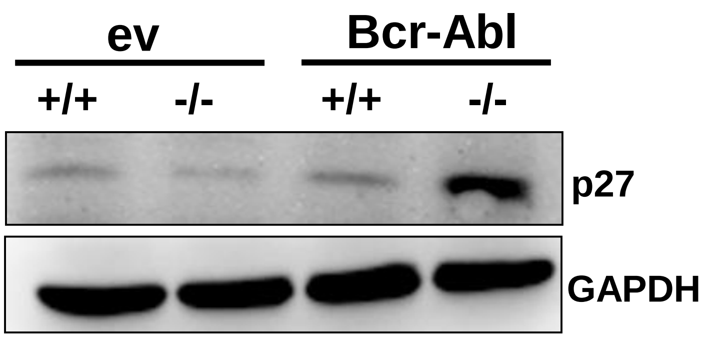

Increased expression of negative cell cycle regulator p27 in Bcr-Abl^{+} cells upon FcγRIIb depletion. p27^{Cip1} protein expression was analyzed by western blot in FcγRIIb$^{+/+}$ and FcγRIIb$^{-/-}$ Bcr-Abl^{+} and empty vector (ev) cells.

Supplementary Figure S6

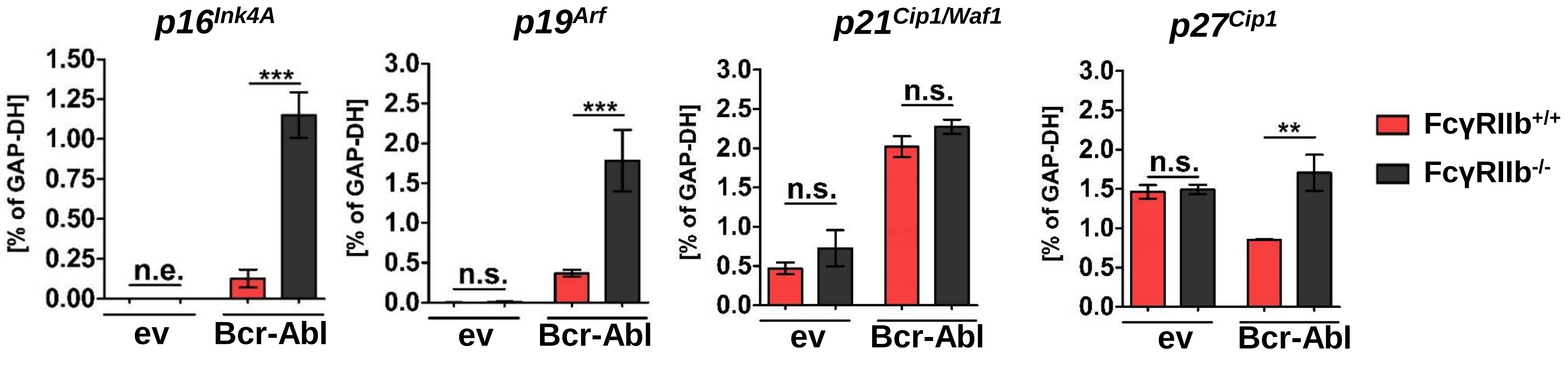

Elevated mRNA levels of negative cell cycle regulators in Bcr-Abl^{+} cells upon FcγRIIb depletion. mRNA expression analyses of p16^{Ink4A}, p19^{Arf}, p21$^{Cip1/Waf1}$ and p27^{Cip1} using qRT-PCR. Data are shown as mean ± SD. n=3 each; **$p<0.001$, ***$p<0.0001$; ns= not statistically significant, ne= not expressed.

Supplementary Figure S7

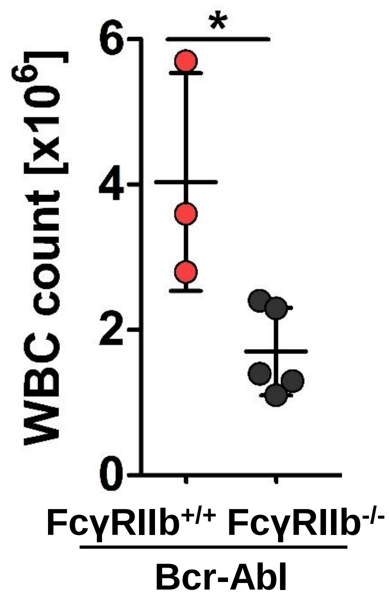

Decreased WBC in Bcr-Abl+ mice upon FcγRIIb depletion. Peripheral blood (PB) was analyzed for white blood cell count (WBC) in transplanted recipients receiving FcγRIIb+/+:Bcr-Abl (n=3) or FcγRIIb-/-:Bcr-Abl (n=5) BM cells. Data are shown as mean ± SD; *p<0.05

Supplementary Figure S8

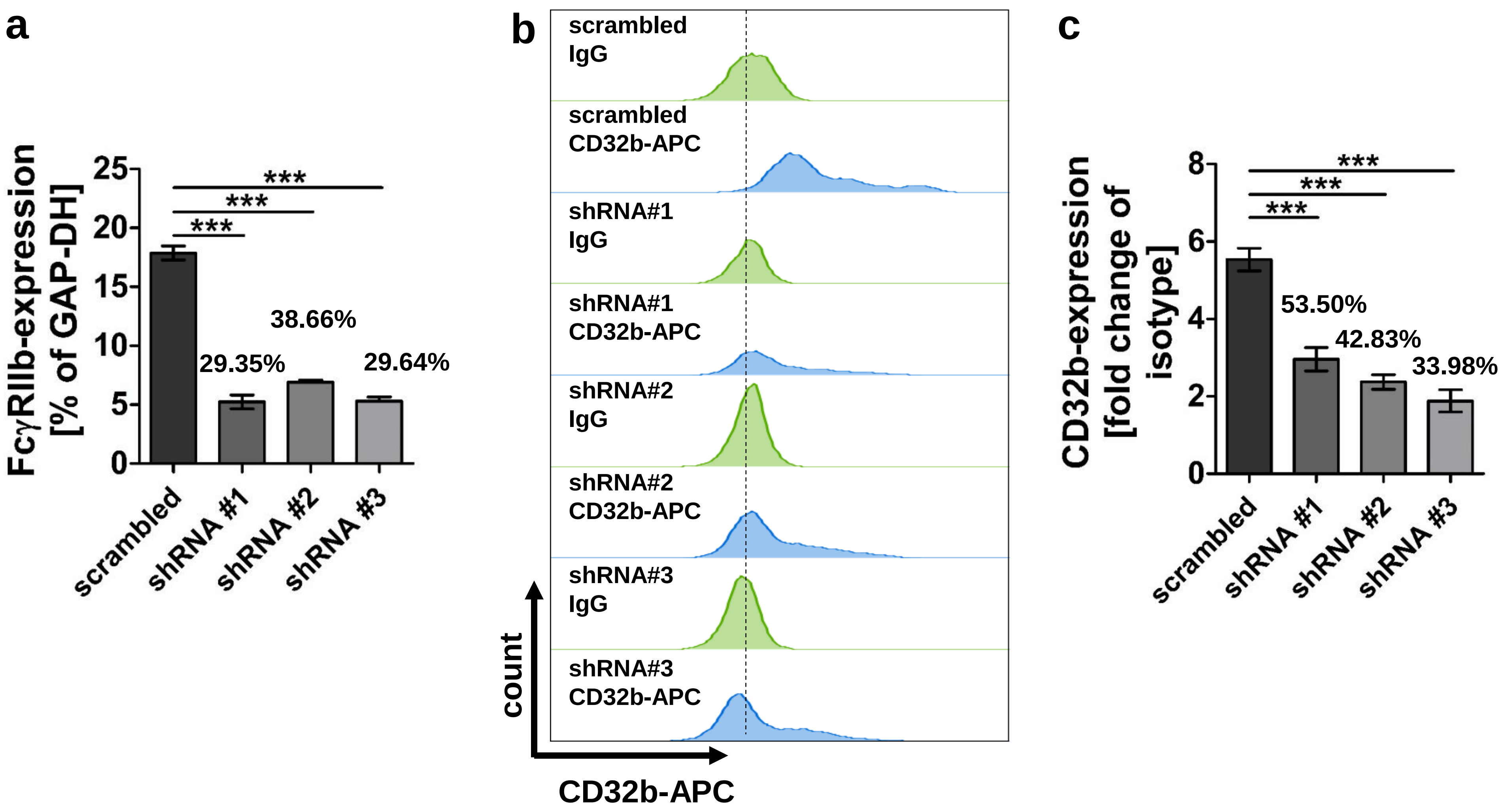

FcγRIIb mRNA and protein expression are significantly reduced by shRNA targeting. Three different shRNAs targeting FcγRIIb were retrovirally introduced into C567B/L6 lin- BM cells. **(a)** FcγRIIb-expression was analyzed using qRT-PCR in FACS sorted infected C567B/L6 lin- BM cells. **(b)** FcγRIIb cell surface expression was analyzed by FACS using CD32b-APC antibody or IgG control. **(c)** Quantification of FcγRIIb surface expression. Data are shown as mean ± SD; ***p<0.0001

Supplementary Figure S9

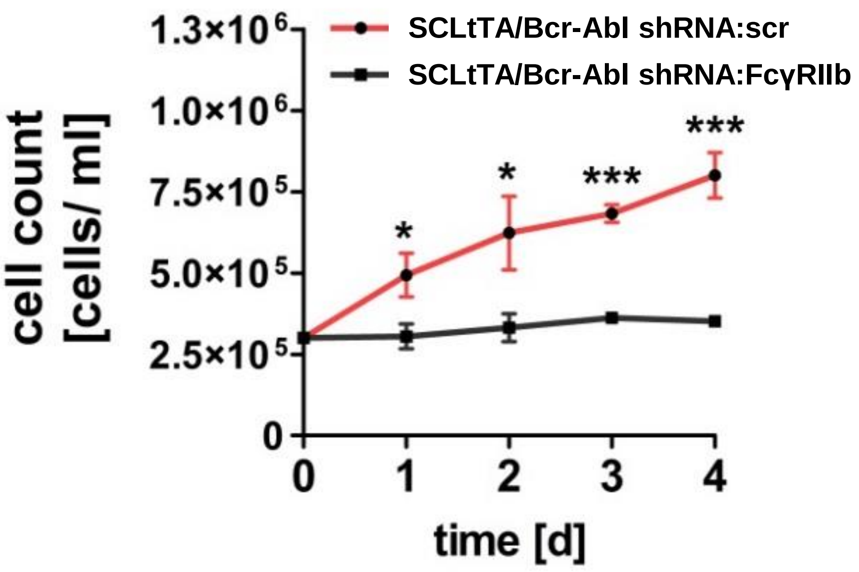

Cell proliferation is decreased in malignant cells upon FcγRIIb targeting. $3x10^5$ cells/ml were used to assess proliferation of immortalized transgenic Bcr-Abl BM cells transduced with shRNA targeting FcγRIIb or scr control (n=3, each).

Supplementary Figure S10

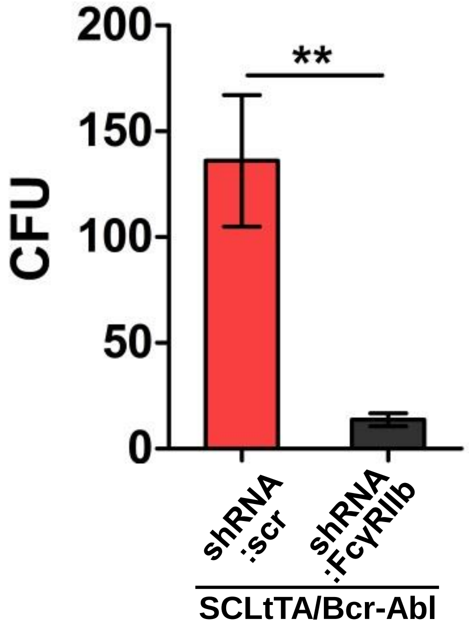

Imparied clonogenic potential in malignant cells upon FcγRIIb targeting. 2000 cells/ml of immortalized SCLtTA/Bcr-Abl cells transduced with shRNA targeting FcγRIIb or shRNA:scr control were seeded in methylcellulose containing cytokines. Colony formation was analyzed after 7 days of culture (n=3, each).

Supplementary Figure S11

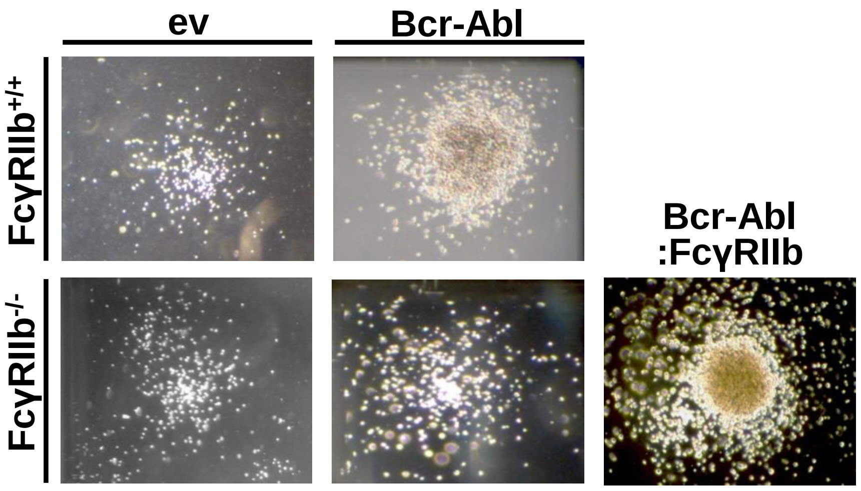

Malignant colony morphology is restored upon FcγRIIb re-expression in malignant cells. CFU morphology of $FcγRIIb^{+/+}$:ev, $FcγRIIb^{-/-}$:ev, $FcγRIIb^{+/+}$:Bcr-Abl, $FcγRIIb^{-/-}$:Bcr-Abl, or $FcγRIIb^{-/-}$:Bcr-Abl:FcγRIIb colonies.

Supplementary Figure S12

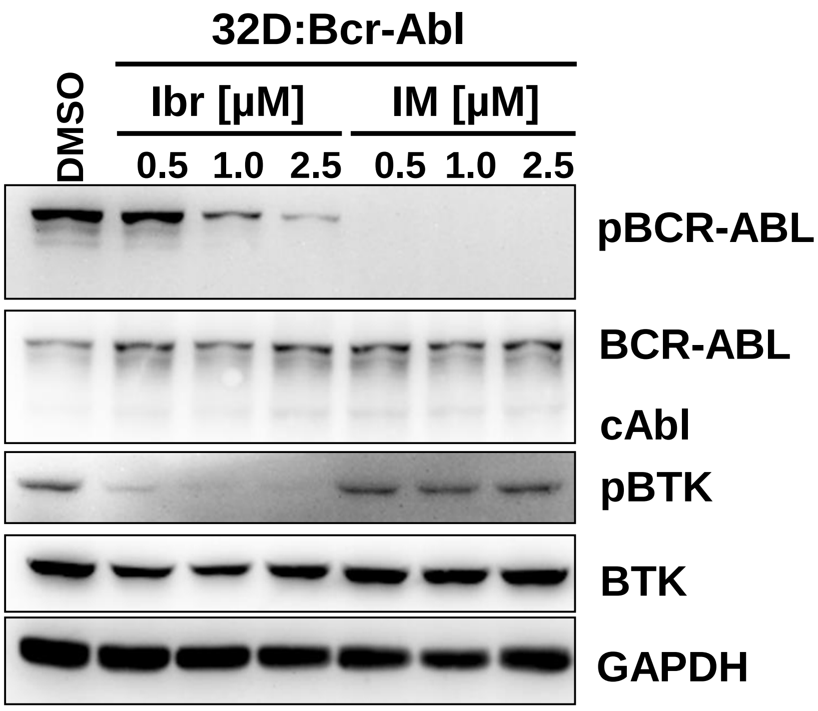

Effect of Ibrutinib treatment on Bcr-Abl kinase activity in 32D:Bcr-Abl cells. 32D:Bcr-Abl cells were subjected to ibrutinib (Ibr, 0.5-2.5 µM) and imatinib (IM, 0.5-2.5 µM) treatment for 4 h. Protein lysates were subsequently analyzed for Bcr-Abl and BTK phosphorylation.

Supplementary Figure S13

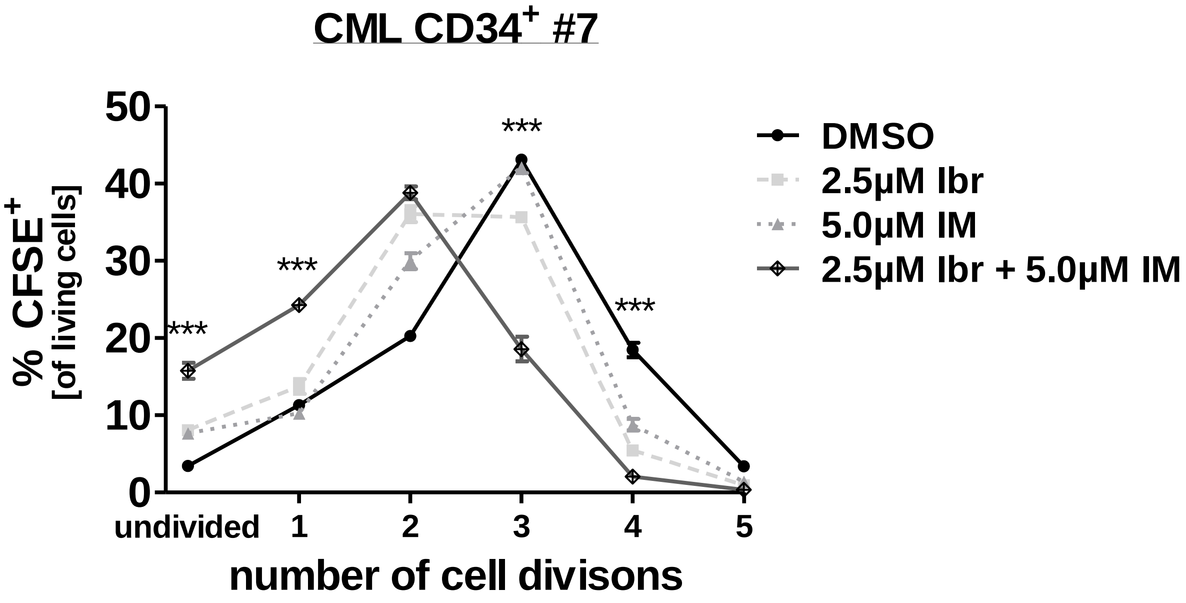

Combined inhibition of Bcr-Abl and BTK reduces cell division in CML CD34⁺ cells (patient CML#4). Evaluation of cell division upon Ibr and IM treatment using CFSE staining. Data are shown as mean ± SD. ***p<0.0001; for a clear presentation, the level of significance is given for Ibr+IM vs. IM only.

Supplementary Figure S14

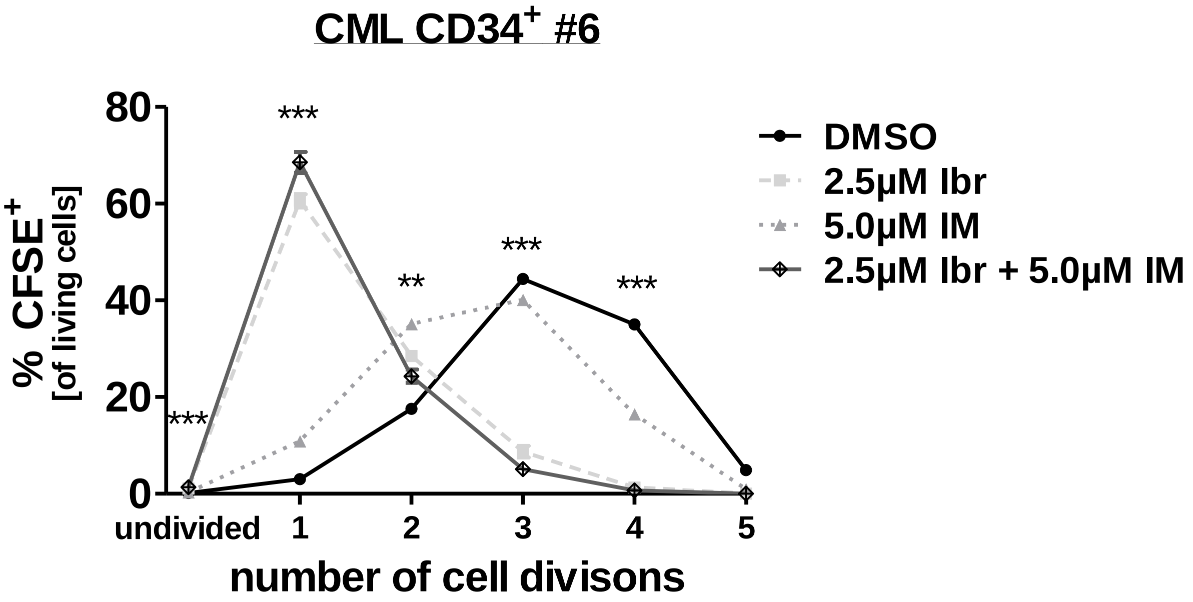

Combined inhibition of Bcr-Abl and BTK reduces cell division in CML CD34⁺ cells (patient CML#4). Evaluation of cell division upon Ibr and IM treatment using CFSE staining. Data are shown as mean ± SD. ***p<0.0001; for a clear presentation, the level of significance is given for Ibr+IM vs. IM only.

Supplementary Figure S15

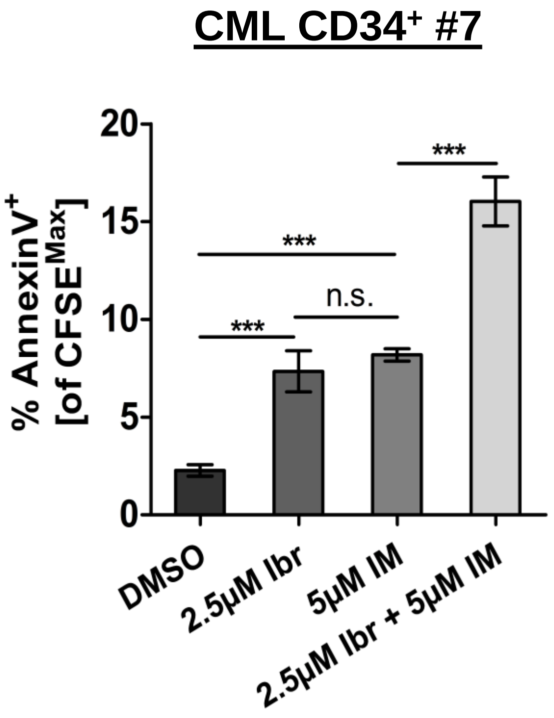

Combined inhibition of Bcr-Abl and BTK enhances apoptosis in non-dividing LSCs of patient #7. AnnexinV$^+$ staining of undivided CFSE^{Max} cells of treated CML CD34$^+$ cells. Data are shown as mean ± SD. ***$p<0.0001$; ns= not statistically significant, Ibr= ibrutinib, IM= imatinib.

Supplementary Figure S16

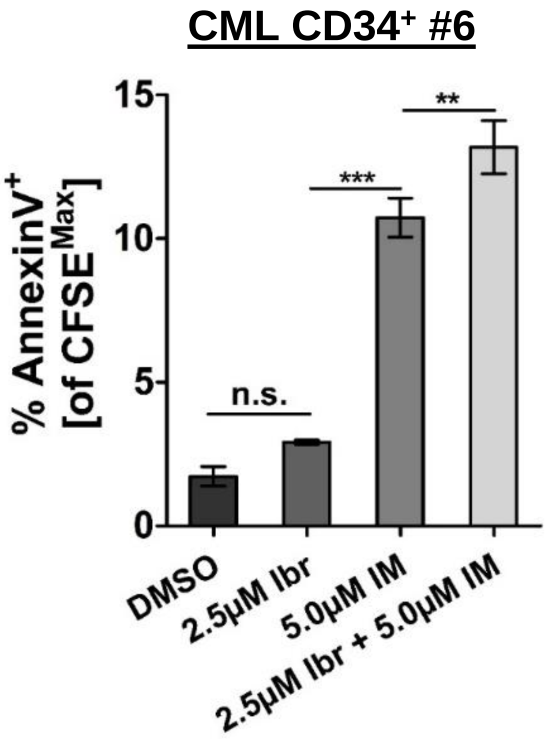

Combined inhibition of Bcr-Abl and BTK enhances apoptosis in non-dividing LSCs of patient #6. AnnexinV$^+$ staining of undivided CFSE^{Max} cells of treated CML CD34$^+$ cells. Data are shown as mean ± SD; **$p<0.001$, ***$p<0.0001$, ns= not statistically significant, Ibr= ibrutinib, IM= imatinib.

Supplementary Figure S17

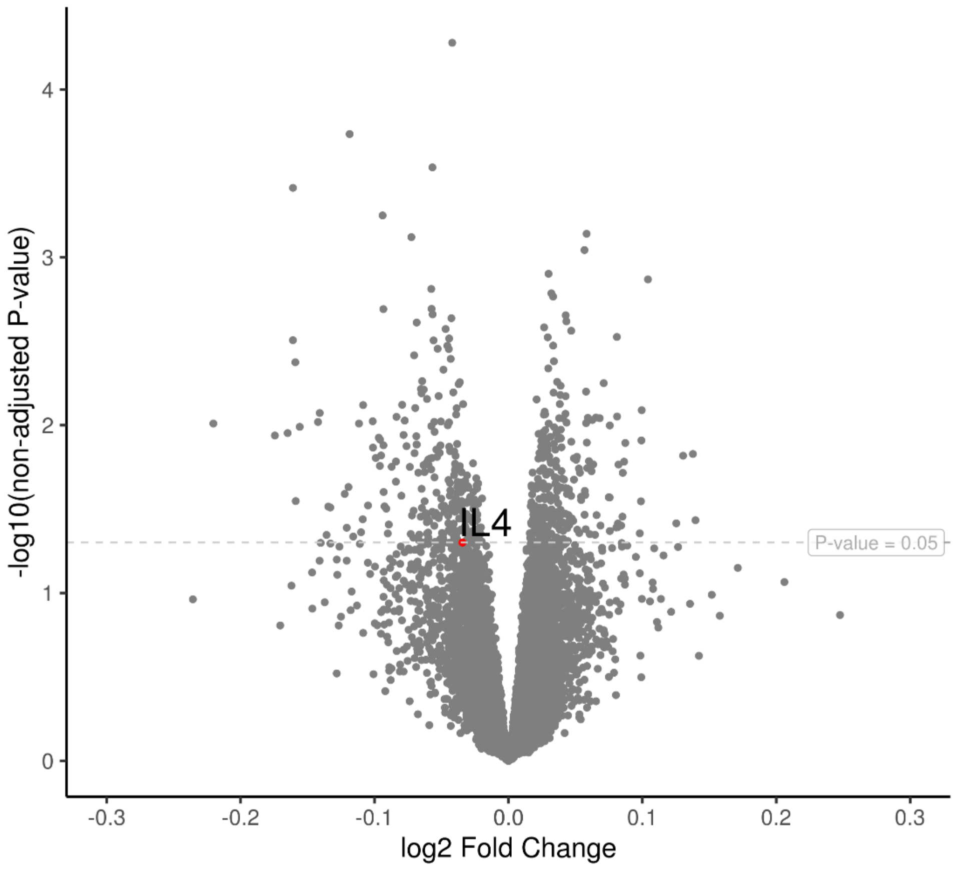

Volcano plot showing gene expression in total BM and pB unfractionated leukocytes in cytogenetic responders compared with non-responders (GSE2535) using non-adjusted p-value.
