# Supplementary material for: Therapeutic inhibition of FcγRIIb signaling targets leukemic stem cells in chronic myeloid leukemia
Source: Leukemia. 2020 Jul 20;34(10):2635–47. doi: 10.1038/s41375-020-0977-8 (PMC7515845; doi:10.1038/s41375-020-0977-8)
Supplement: Supplementary file 2 — Supplementary Information Tables [file 41375_2020_977_MOESM2_ESM.pdf]

Supplementary Information

Therapeutic inhibition of FcγRIIb signaling targets leukemic stem cells in chronic myeloid leukemia

Oliver Parting[1], Samantha Langer[2], Maja Kim Kuepper[1], Caroline Wessling[3], Shaoguang Li[4], Till Braunschweig[5], Nicolas Chatain[1], Michael Huber[6], Tim H Brümmendorf[1], Steffen Koschmieder[1], and Mirle Schemionek[1]

[1]Deptartment of Hematology, Oncology, Hemostaseology, and Stem Cell Transplantation, Faculty of Medicine, University Hospital RWTH Aachen, Aachen, Germany; [2]Department of Transfusion Medicine, University Hospital Essen, Essen, Germany; [3]Department of Neurosurgery, Clemens Hospital, Muenster, Germany; [4]Department of Medicine, University of Massachusetts Medical School, Worcester, Massachusetts, USA; [5]Department of Pathology, University Hospital RWTH Aachen, Aachen, Germany; [6]Institute of Biochemistry and Molecular Immunology, RWTH Aachen University, Aachen, Germany

Supplementary Table S1: sequences of primer and probes applied in this study

Oligonucleotide	Sequence 5'-3'
Bcr-Abl forward	CGTCAACTCAGCCACTGG
Bcr-Abl reverse	GGCTTCACTCAGACCCTGA
Bcr-Abl probe	FAM-AGCGGCCAGTATCATCTGACTTTGAGC-TAMRA
GAPDH forward	TTGTGCAGTGCCAGCCTC
GAPDH reverse	CCAATACGGCCAATCCG
GAPDH probe	FAM-TCCCGTAGACAAAATGGTGAAGGTCGGT-TAMRA
p16 forward	TCATCATCACCTGAATCGGGG
p16 reverse	AAAGCGAACTCGAGGAGAGC
p19 forward	GCCGCACCGGAATCCT
p19 reverse	TTGAGCAGAAGAGCTGCTACGT
p21 forward	GTGGGTCTGACTCCAGCCC
p21 reverse	CCTTCTCGTGAGACGCTTAC
p27 forward	TCGCAAAACAAAAGGGCCAA
p27 reverse	CTTAATTCGGAGCTGTTTACGTC

Supplementary Table S2: shRNAs sequences

shRNA	Sequence 5'-3'
#1_shRNA_ FcγRIIb_F	TCGAGTGCTGTTGACAGTGAGCGCCGGTGACACTGAC ATGCGAAGTAGTGAAGCCACAGATGTACTTCGCATGT CAGTGTCACCGTTGCCTACTGCCTCGGAG
#1_shRNA_ FcγRIIb_R	AATTCTCCGAGGCAGTAGGCAACGGTGACACTGACAT GCGAAGTACATCTGTGGCTTCACTACTTCGCATGTCA GTGTCACCGGCGCTCACTGTCAACAGCAC
#2_shRNA_ FcγRIIb_F	TCGAGTGCTGTTGACAGTGAGCGCGGGATGCTGTAGA TATTAAAGTAGTGAAGCCACAGATGTACTTTAATATCTA CAGCATCCCTTGCCTACTGCCTCGGAG
#2_shRNA_ FcγRIIb_R	AATTCTCCGAGGCAGTAGGCAAGGGATGCTGTAGATA TTAAAGTACATCTGTGGCTTCACTACTTTAATATCTACA GCATCCCGCGCTCACTGTCAACAGCAC
#3_shRNA_ FcγRIIb_F	TCGAGTGCTGTTGACAGTGAGCGAGGATGCTGTAGAT ATTAAAGATAGTGAAGCCACAGATGTATCTTTAATATCT ACAGCATCCCTGCCTACTGCCTCGGAG
#3_shRNA_ FcγRIIb_R	AATTCTCCGAGGCAGTAGGCAGGGATGCTGTAGATAT TAAAGATACATCTGTGGCTTCACTATCTTTAATATCTAC AGCATCCTCGCTCACTGTCAACAGCAC
Scrambled_ FcγRIIb_F	TCGAGTGCTGTTGACAGTGAGCGCGGTAAATTATTGT GATTTAGTTAGTGAAGCCACAGATGTAACTAAATCACA ATAATTTACCTTGCCTACTGCCTCGGAG
Scrambled_ FcγRIIb_R	AATTCTCCGAGGCAGTAGGCAAGGTAAATTATTGTGAT TTAGTTACATCTGTGGCTTCACTAACTAAATCACAATAA TTTACCGCGCTCACTGTCAACAGCAC

Supplementary Table S3: Antibodies for immunophenotyping applied in this study

Antibody	Supplier	Cell clone
CD3-PE Cy5	Biolegend	17A2
Gr-1-PE Cy5	Biolegend	RB6-8C5
Gr-1-Tri Color	Life technologies	RM3006
CD4-Tri Color	Life technologies	MCD0406
CD4-PE Cy5	Biolegend	RM4-5
CD8α-Tri Color	Life technologies	MCD0806
CD8a-PECy5	Biolegend	53-6.7
CD11b-PE Cy5	Biolegend	M1/70
CD117(cKit) -APC Cy7	Biolegend	2B8
Sca-1-Biotin	Biolegend	E12-161.7
Streptavidin-PE Cy7	BD Bioscience	
CD45.1-APC	Biolegende	A20
CD45.2-Pacific Blue	Biolegende	104
CD32b-PE	eBioscience	AT130-2
CD32b-APC	eBioscience	AT130-2
Rat IgG2a kappa-PE	eBioscience	eBR2a
Rat IgG2a kappa-APC	eBioscience	eBR2a
